# Whole genome sequencing data analysis identified a cefotaxime-resistant *Empedobacter brevis* GBW-1 isolate from ground beef encoding a novel metallo-beta-lactamase variant, *blaEBR-6*

**DOI:** 10.1016/j.dib.2026.112547

**Published:** 2026-02-06

**Authors:** Daniel Jones, Praful Aggarwal, Jamison Trewyn, Poojhaa Shanmugam, Kyle Leistikow, Troy Skwor

**Affiliations:** a Department of Biomedical Science, University of Wisconsin- Milwaukee, Enderis Hall, 2400 E. Hartford Ave., Milwaukee, WI 53211, USA; b Microbial Discovery Group, 7420 S. Howell Ave. Suite 300, Oak Creek, WI 53154, USA; c Eurofins Microbiology Laboratories Inc., 2345 S. 170th St., New Berlin, WI 53151, USA

**Keywords:** Antimicrobial resistance genes (ARGS), Foodborne bacteria, EBR, Ambler Class B, Meat, Carbapenemase

## Abstract

While investigating foodstuffs for ESBL-producing *Aeromonas* species on ampicillin dextrin agar with vancomycin and cefotaxime, a multidrug-resistant *Empedobacter brevis* strain GBW-1 was identified from ground beef. Phylogenetic analysis supports the interconnectedness of environment, humans and food driving this species' evolutionary development. Antimicrobial susceptibility testing demonstrated resistance to gentamicin, carbapenems and third-generation cephalosporins. Data collection from whole genome sequencing of this strain detected a 3.74 Mb genome with 32.8% GC content containing 3780 coding genes. Among these genes, at least three known antimicrobial resistance (AMR) genes were identified from the dataset with *qacG, vanT* gene within the *vanG* cluster, and a novel variant of the metallo-β-lactamase *bla*_EBR-6_. This homologue, EBR-6, was compared against previously known EBR variants and was found to be closest to EBR-3 with an 84.98% amino acid identity match. Data collection from *in silico* molecular docking experiments predicted these mutations change the binding to meropenem. Furthermore, nearly 100 annotated regions associated with mobile genetic elements, including the presence of *tra* operons, were identified on the genome. Together, this dataset provides, genomic, phenotypic, and *in* silico data that may be reused to monitor the evolution of EBR from a One Health perspective.

Specifications Table

**Table:** 

Subject	Biology
Specific subject area	Microbiology, bacteriology, molecular biology, genomics, infectious diseases
Type of data	Tables, Figures, including Supplemental Figures and Tables showing whole genome sequence and predicted binding of EBR-6 to meropenem
Data collection	*Empedobacter brevis* GBW-1 isolated from ground beef and subjected to antimicrobial susceptibility and genomic analysis. The WGS data of *Empedobacter brevis* GBW-1 was performed on the Illumina NextSeq2000 platform (2 × 150 bp paired reads). Genomic assembly and annotation were performed via the Unicycler v0.4.8 and RAST toolkit (RASTtk) through BV-BRC. Putative mobile genetic elements were predicted via mobileOG-db v1.1.3 and ran on the Proksee web server. AlphaFold3 was used for computational 3D modeling of a new EBR variant, EBR-6. Furthermore, molecular docking of the new EBR variant to meropenem were predicted using BIOVIA Discovery Studio Visualizer and AutoDock4.
Data source location	University of Wisconsin – Milwaukee, School of Biomedical Sciences and Health Care Administration, Milwaukee, Wisconsin, USA
Data accessibility	Repository name: EBR-6 Empedobacter brevis raw data [1]; NCBI GenBank Accession# JBLTIN000000000 Data identification number: DOI: 10.17632/7gn49wxtrr.1 Direct URL to the dataset, which is organized by figure folders, containing raw sequence files, EBR comparison to other ESBLs and difference in protein folding, and files use to compute EBR-meropenem docking is at: https://data.mendeley.com/datasets/7gn49wxtrr/1
Related research article	None.

## 1 Value of the Data

• This data provides genomic insight into multi-drug resistant (MDR) bacterial populations such as *Empedobacter brevis* which is considered an emerging opportunistic pathogen.
• The dataset presented here provides the first report of a novel metallo-β-lactamase (*bla*_EBR-6_) variant identified in an *Empedobacter brevis* isolated from ground beef.
• This data report expands the understanding of how foodborne bacteria may serve as reservoirs and vectors for the further spread of resistance genes and reinforces the necessity of utilizing a collaborative One Health approach to combat AMR across sectors.

## 2 Background

In 2024, carbapenem- and third-generation cephalosporin-resistance among *Enterobacterales* remained in the ‘critical’ group of urgency among drug resistant priority pathogens reported by the World Health Organization [2]. Due to their ubiquity within wide-ranging environments, capability of intra- and inter-species conjugation, and a growing repertoire of antibiotic resistance genes (ARGs), the Gram-negative *Aeromonas* bacteria have been implicated as a possible indicator species for One Health antimicrobial resistance (AMR) surveillance [3]. However, AMR within food among *Aeromonas* is limited and to help bridge this gap, we sought out ESBL-producing *Aeromonas* spp. in food. After identifying a yellow colony on ampicillin dextrin agar with cefotaxime, confirmatory biochemical tests revealed it as oxidase and indole positive, but negative on trehalose, indicating it was not an *Aeromonas* isolate. Subsequent whole-genome sequencing identified this strain as *Empedobacter brevis* GBW-1. Here we aimed to characterize this strain with a focus on its genomic features and a novel metallo-β-lactamase gene variant, *bla*EBR-6.

## 3 Data Description

### 3.1 Antimicrobial susceptibility and genome annotation

**Fig. 1:**
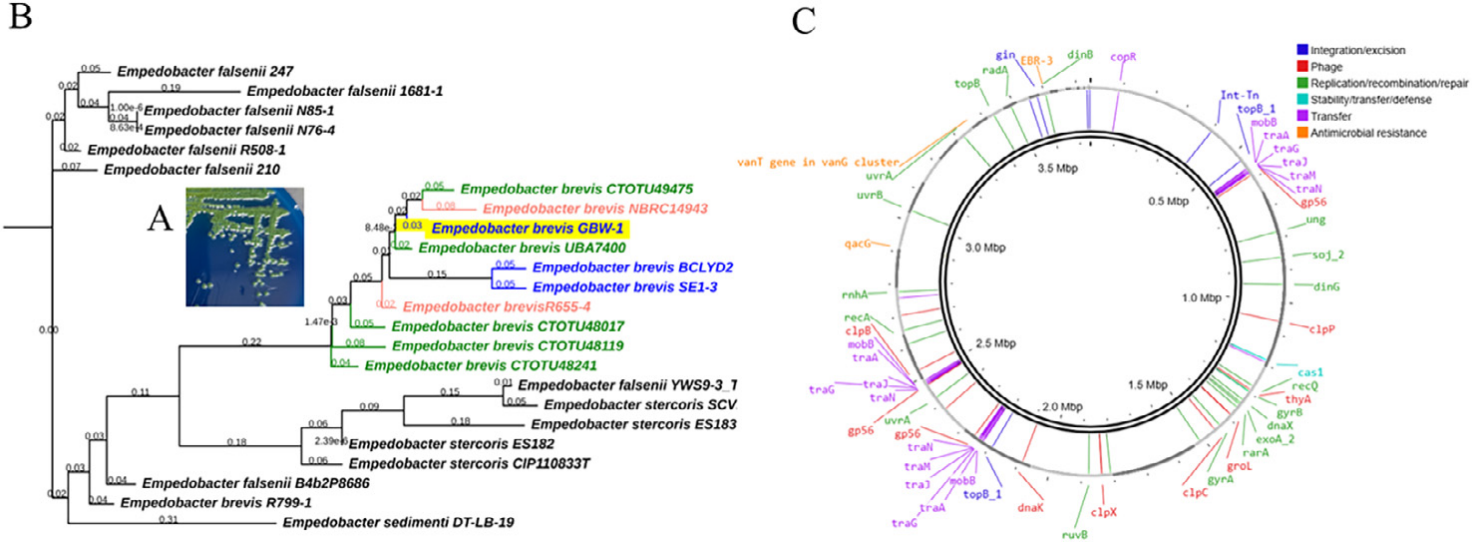
Isolation and whole genome analysis of *Empedobacter brevis* GBW-1. (A) *Empedobacter brevis* GBW-1 colonies on ADA-V agar subsequent to 24 h incubation at 30 °C. (B) Phylogenetic tree of *Empedobacter* genomes retrieved from NCBI. Strain GBW-1 (highlighted) is clustered with other *E. brevis* strains. Human isolates of *E. brevis* are in pink, while environmental isolates are in green and food isolates in blue. (C) Genetic map of *E. brevis* GBW-1 showing putative sites for ARGs along with the annotated protein families mediating the integration/excision, replication/recombination/repair, stability/transfer/defense or transfer of bacterial mobile genetic elements and phages.

Fig. 1A displays the morphologic features of *Empedobacter brevis* GBW-1 on ampicillin dextrin agar with vancomycin and cefotaxime (Fig. 1A) isolated from ground beef samples. Antimicrobial susceptibility testing identified resistance to gentamicin, third-generation cephalosporins (cefotaxime and ceftazidime) with and without beta-lactamase inhibitors, and meropenem (Suppl. Table 1).

**Table 1:** Summary of genomic features of *E. brevis* GBW-1.

Parameter	
Organism Bases (Mb) Contig N50 Contigs Completeness ( %) Contamination ( %) GC content ( %)	*Empedobacter brevis* 3.74 254,112 39 100.0 0.0 32.81
CDS	3629
rRNA	3
tRNA ARGs MGEs	65 3 95

Whole genome analysis displayed a complete genome assembly with a size of 3.74 Mb with 32.8 % GC content that encoded for 3780 protein-coding genes (Table 1). Phylogenetic analysis of GBW-1 with other environmental, food, and human *E. brevis* isolates was represented in Fig. 1B The whole genome of *E. brevis* GBW-1 was predicted to have 3629 coding regions with 65 tRNA and 3 rRNAs. Over 95 mobile genetic elements (Suppl. Table 2) were identified as well as the presence of genes associated with capsule formation, iron uptake, anti-oxidants, and type 4 and 6 secretion systems (Supplemental Table 3).

Fig. 1C shows a genetic map of *Empedobacter brevis* GBW-1. Three antimicrobial resistance genes were identified: *vanTG* (vanT gene in vanG cluster), *qacG*, and a *bla*_EBR._.

### 3.2 Identification and characterization of novel metallo-β-lactamase, EBR-6

**Fig. 2:**
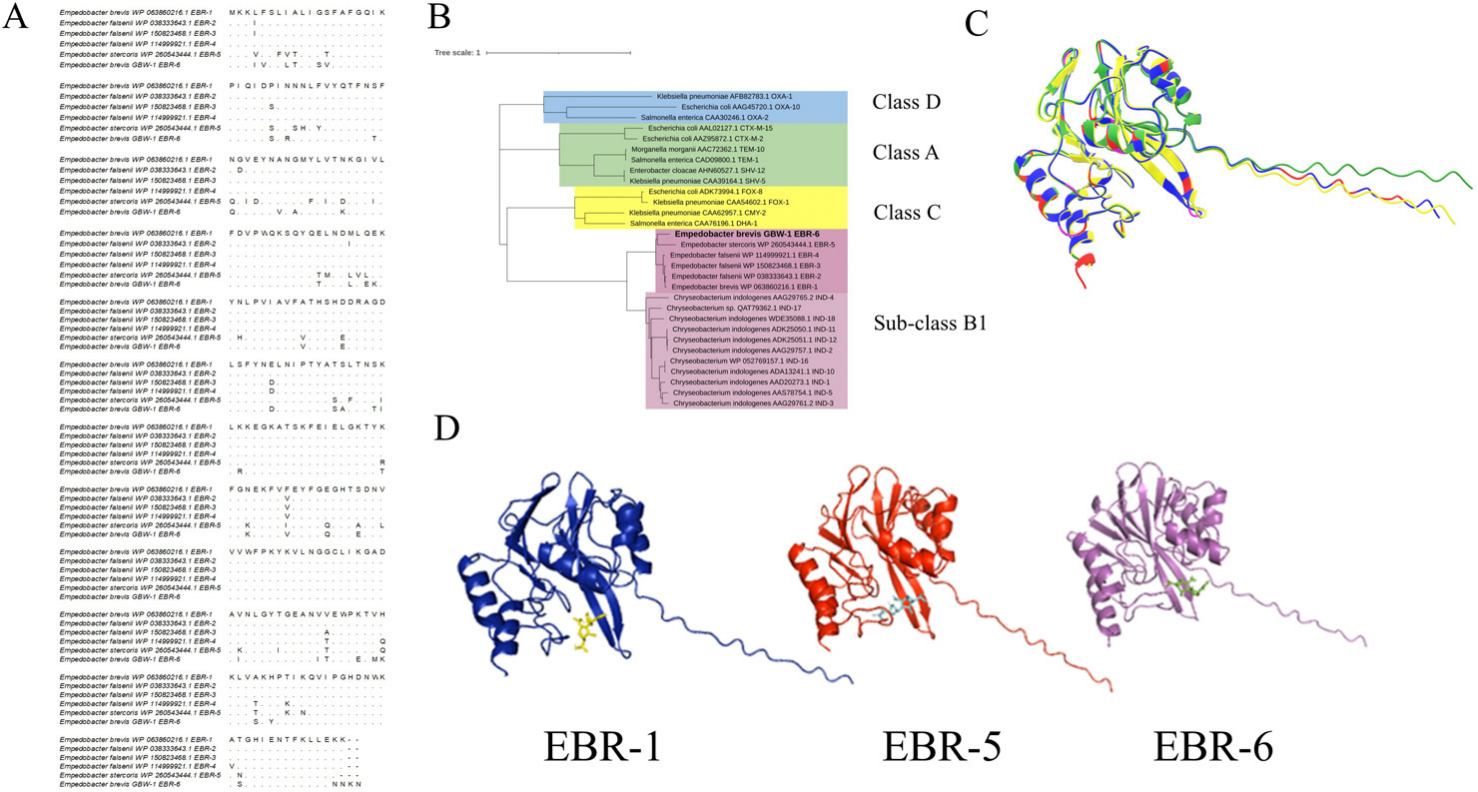
Phylogenetic analysis and variance of protein structure and meropenem docking among EBRs. (A) Amino acid comparison between all known EBRs. (B) Phylogenetic tree comparing amino acid sequences of EBR-6 to other beta-lactamases. (C) Comparison of predicted 3D protein structure of EBR-6 (blue) to EBR-1 (yellow) and EBR-5 (green). Amino acids differing between all three proteins are represented in red whereby differences between EBR-1 and -6 are in pink. (D) Computational predictions of meropenem docking between EBR-1, -5 and -6.

CARD and BLAST (BLASTP 2.12.0 on 5/16/2025) analysis of the predicted amino acid sequence of EBR revealed 84.98 % similarity to its closest relative, EBR-3 (Fig. 2A) isolated from a human rectal swab (Suppl. Table 4), indicating this gene represents a previously undesignated variant, herein designated *bla*_EBR-6_ (Accession# MGU9940303.1). Confirmation of the presence of a metallo-β-lactamase in *E. brevis* GBW-1 was assessed by EDTA inactivation of carbapenemase activity against meropenem exhibited Suppl. Table 5 and Suppl. Fig. 1). From an evolutionary perspective, EBR-5 is the most recent descendent (Fig. 2B) using the neighbor-joining method through MEGA11 [4]. EBR-5 was identified in *Empedobacter stercoris* from chicken feces in China (Suppl. Table 4) and is 81.1 % similar at the amino acid level.

Due to the similarity to EBR-5, computational 3D renderings using AlphaFold3 [5] and ChimeraX 1.9 [6] were used to predict and compare EBR-1, EBR-5, and EBR-6 (Fig. 2C). The 5 models constructed by AlphaFold3 were validated for quality using ProSA-web (Z-scores) [7]. All the models have a Z-score of –8 and fall under the NMR region indicating significant quality (Suppl. Fig. 2). The best binding poses from each of the EBR-meropenem complexes were selected based on lowest binding affinity (kcal/mol) (Fig. 2D and Suppl. Table 6). Predicted common strong interactions (i.e. hydrogen, pi-sigma, pi-alkyl) with meropenem for the three EBR proteins were at Phe40, Tyr186, and His216 (Suppl. Figure 3 and 4). Overall, EBR-5 and -6 both have more hydrogen and pi-bonds than EBR-1 (Suppl. Figure 3 and 4).

## 4 Experimental Design, Materialsand Methods

### 4.1 Bacterial isolation and identification

Ground beef samples (25 g) were homogenized in 0.1 % (w/v) peptone and plated on ampicllin dextrin agar supplemented with vancomycin and cefotaxime (1µg/ml) (ADA-V) before incubating overnight at 30 °C. The subsequent day, yellow colonies (Fig. 1A) were selected as presumptive *Aeromonas* spp. and further characterized. Biochemical tests (i.e. oxidase, indole, and trehalose) were performed to further identify the species, with oxidase and indole tests providing positive results. However, the negative trehalose test demonstrated the bacteria was not *Aeromonas* and the bacterial genome was sent for sequencing for identification.

### 4.2 DNA extraction and sequencing

Genomic DNA was extracted from overnight liquid tryptic soy broth (TSB) culture started from a -80 °C frozen glycerol stock, using the Wizard Genomic DNA Purification Kit (Promega) and quantified via Qubit (Thermo Fisher Scientific) following manufacturer protocols. Genomic libraries were prepared using the Illumina DNA Prep fragmentation kit (Illumina, Inc., San Diego, CA, USA) and Illumina Unique Dual Indexes prior to whole genome sequencing on the Illumina NextSeq2000 platform (2 × 150 bp paired reads). Genomic assembly and annotation were submitted for the comprehensive genome analysis service at BV-BRC [8] and submitted to NCBI (Accession# JBLTIN000000000). Genome assembly was performed using Unicycler v0.4.8 and annotation was performed with RAST toolkit (RASTtk) through BV-BRC. Genome Quality was assessed using CheckM [9] via KBase [10]. Taxonomic classification was performed using Taxonomic database toolkit, GTDB-Tk v2.4.0 [11]. Antimicrobial genes were detected using the comprehensive antibiotic resistance database (RGI 6.0.5, CARD v4.0.1) on July 11, 2025. Putative mobile genetic elements were predicted using mobileOG-db v1.1.3 [12] run on the Proksee web server [13]. To further assess the potential of *E. brevis* to be pathogenic Virulence Factor Database (VFDB) was used to identify putative virulence factors [14].

### 4.3 mCIM and eCIM to identify metallo-β-lactamase activity

Modified carbapenem inactivation methods (mCIM) and EDTA-modified carbapenem inactivation method (eCIM) assays were performed simultaneously to verify metallo-β-lactamase activity of EBR-6 following a previous study [15]. Control strains were obtained from the Centers for Disease Control and Prevention (CDC) and U.S. Food and Drug Administration (FDA) Antibiotic Resistance Isolate Bank (ARB) [16]. *Pseudomonas aeruginosa* (ARB0103) which encodes for the metallo-β-lactamase, *bla*_IMP-1_, as well as *Enterobacter cloacae* (ARB0053) which encodes for the serine carbapenemase, *bla*_KPC-3,_ were used as control strains for comparison against *E. brevis* GBW-1. Presence of a metallo-β-lactamase was confirmed if the zone of inhibition on the eCIM disk was ≥ 5 mm compared to the mCIM. To further confirm the inhibitory effect of EDTA on carbapenemase activity, 20 µl of 100 mM EDTA was added in a 6 mm spot immediately to the left of a MEM disk (Suppl. Fig. 1B) or left with just meropenem (Suppl. Fig. 1A) subsequent to spreading a 0.5 McFarland of *E. brevis* GBW-1 over the plate following the Clinical and Laboratory Standards Institute (CLSI) M100 antimicrobial susceptibility disk diffusion assay protocol.

### 4.4 EBR-6 in silico modeling

Computational 3D renderings using AlphaFold3 [5] and ChimeraX 1.9 [6] were used to predict and compare EBR-1, EBR-5, and EBR-6 (Fig. 2B). The 5 models constructed by AlphaFold3 were validated for quality using ProSA-web (Z-scores) [7]. The lowest Z-score EBR models and meropenem were cleaned, charged and hydrogenated for docking in BIOVIA Discovery Studio Visualizer [17] and AutoDock4 [18]. Molecular docking was carried out using AutoDock Vina [19]. The best binding poses from each of the EBR-meropenem complexes were selected based on lowest binding affinity (kcal/mol), suggesting strongest predicted binding (Suppl. Table 2). The docking interactions were analyzed using BIOVIA to identify types of molecular interactions (hydrogen bonds or hydrophobic contacts) caused by the ligand (Suppl. Fig. 2). PyMOL [20] was used to estimate the differences in the binding of meropenem with each of the EBR proteins (Fig. 2C).

## Limitations

None.

## Ethics Statement

No human or animal subjects were used in this study. The authors declare that this manuscript follows all ethical requirements of publication in Data in Brief and is original work that has not been published elsewhere.

## CRediT authorship contribution statement

**Daniel Jones:** Data curation, Formal analysis, Investigation, Methodology, Software, Validation, Visualization, Writing – original draft, Writing – review & editing. **Praful Aggarwal:** Data curation, Formal analysis, Investigation, Methodology, Resources, Visualization, Writing – review & editing. **Jamison Trewyn:** Data curation, Formal analysis, Funding acquisition, Investigation, Methodology, Resources, Validation, Writing – review & editing. **Poojhaa Shanmugam:** Investigation, Methodology, Software, Visualization, Writing – review & editing. **Kyle Leistikow:** Investigation, Project administration, Resources, Supervision, Validation, Writing – review & editing. **Troy Skwor:** Conceptualization, Data curation, Formal analysis, Funding acquisition, Investigation, Methodology, Project administration, Resources, Supervision, Validation, Visualization, Writing – original draft, Writing – review & editing.

## Data Availability

Mendeley DataEBR-6 Empedobacter brevis raw data (Original data).

## Appendix Supplementary materials

Image, application 1
