# Supplementary material for: Whole genome sequencing data analysis identified a cefotaxime-resistant Empedobacter brevis GBW-1 isolate from ground beef encoding a novel metallo-beta-lactamase variant, blaEBR-6
Source: Data Brief. 2026 Feb 6;65:112547. doi: 10.1016/j.dib.2026.112547 (PMC12925461; doi:10.1016/j.dib.2026.112547)
Supplement: Supplementary file 1 [file mmc1.docx]

**Supplemental Figures**


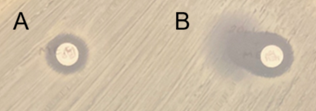


Supplemental Figure 1. Effect of EDTA on *Empedobacter brevis* GBW-1 carbapenemase activity. Meropenem disks were added to a plate of *E. brevis* GBW-1 with (B) or without EDTA (A) immediately plated to the left of the disk. Zone of inhibitions of MEM alone (A) is 12 mm and with EDTA (B) is 19mm.

EBR1


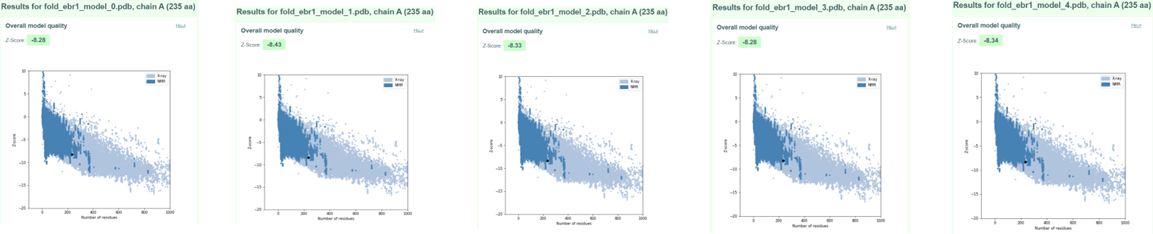


EBR5


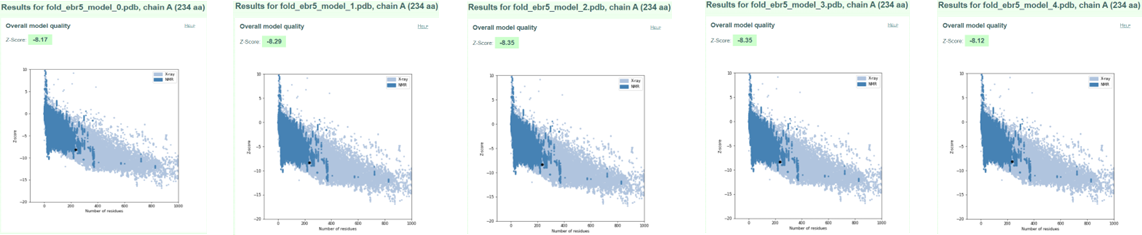


EBR6


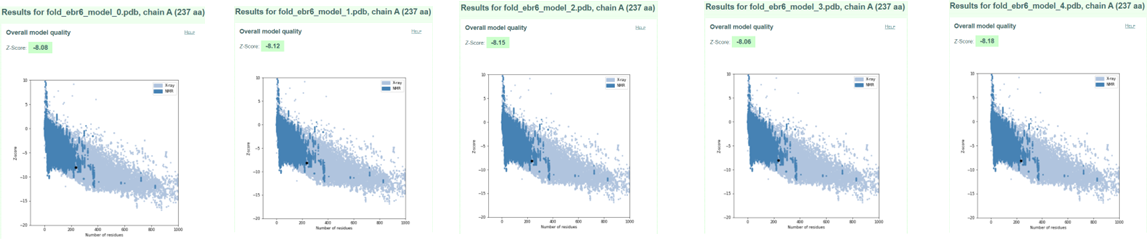


**Supplemental Figure 2. Predicted structure validation of EBRs**

EBR1


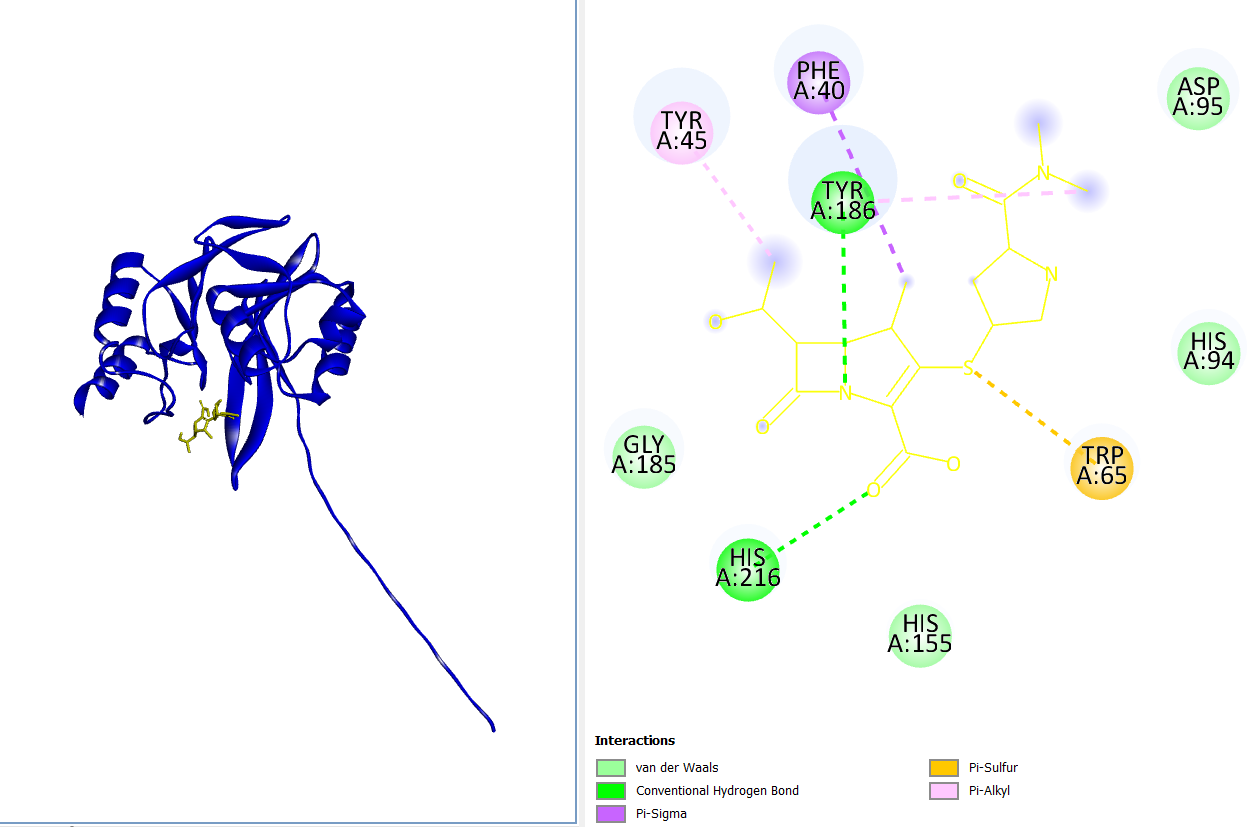


EBR5


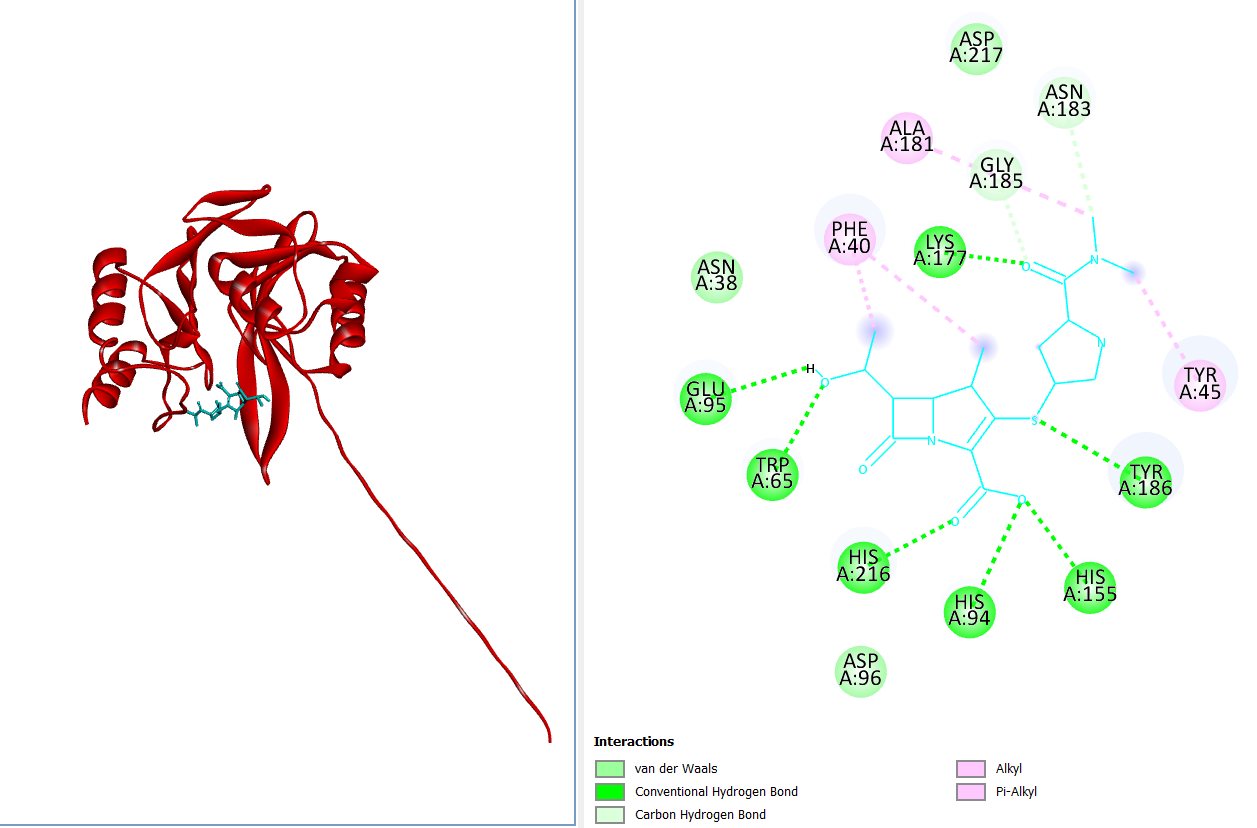


EBR6


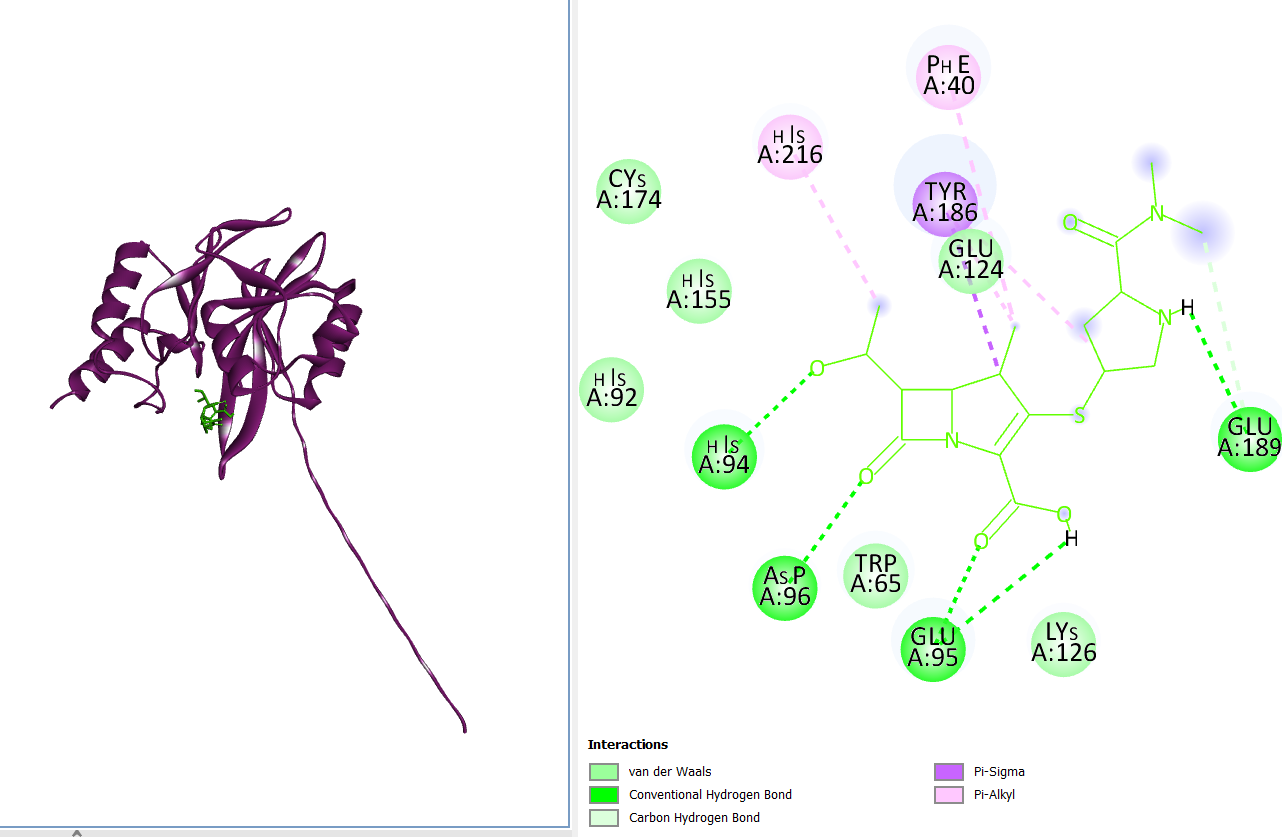


**Supplemental Figure 3. Predicted docking interactions of EBR with meropenem**


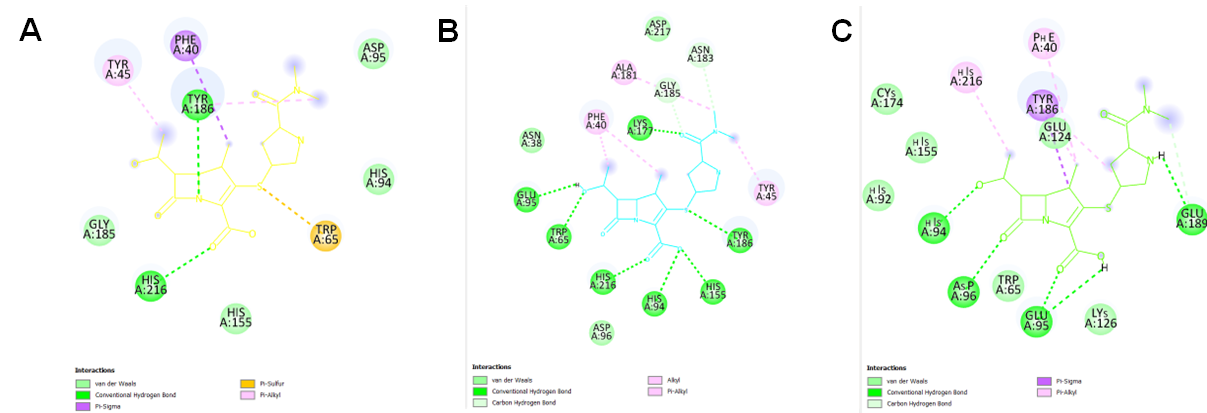


**Supplemental Figure 4. Docking interaction of EBR with meropenem.** (A) EBR1-meropenem, (B) EBR5-meropenem, (C) EBR6-meropenem

**Supplemental Tables**

Supplemental Table 1. Antimicrobial susceptibility of *E. brevis* GBW-1

| **Antibiotic** | **Zone of inhibition (mm)** | **Susceptiblity/Resistant (S/R)** |
| --- | --- | --- |
| Amoxacillin/Clavulanic acid | 11 | R |
| Cefotaxime | 14 | R |
| Ceftazidime | 17 | R |
| Ceftazidime/clavulanic acid | 16 | R |
| Ceftazidime/avibactam | 13 | R |
| Cefepime | 29 | S |
| Meropenem | 9 | R |
| Aztreonam | 21 | S |
| Azithromycin | 31 | S |
| Sulfamethoxazole/trimethoprim | 21 | S |
| Ciprofloxacin | 23 | S |
| Tetracycline | 18 | S |
| Gentamicin | 11 | R |
| Chloramphenicol | 29 | S |

Supplemental Table 2. Mobile genetic elements predicted from *E. brevis* GBW-1 (e-value < 1.0e-05

| **MGE_name** | **Gene Name** | **mobileOG ID** | **Major mobileOG Category** | **Source Database** | **Evidence Type** | **% identity** | **Bitscore** | **e-value** | **ORF #Start #Stop #Strand** |
| --- | --- | --- | --- | --- | --- | --- | --- | --- | --- |
| mobileOG_000299816_contig_1 | copR | mobileOG_000299816 | transfer | Plasmid RefSeq | Homology | 68.9 | 313.2 | 3.6e-84 | 87176 # 87865 # 1 |
| mobileOG_000237009_contig_1 | Int-Tn | mobileOG_000237009 | integration/excision | GPD | Homology | 68.7 | 453.4 | 3.0e-126 | 402872 # 403795 # 1 |
| mobileOG_000719990_contig_1 | NA:Keyword | mobileOG_000719990 | transfer | ICE | Keyword Search | 87.9 | 509.6 | 3.2e-143 | 527121 # 527966 # 1 |
| mobileOG_000268337_contig_1 | topB_1 | mobileOG_000268337 | integration/excision | ICE | Homology | 95.8 | 1319.3 | 0 | 530468 # 532555 # 1 |
| mobileOG_000719989_contig_1 | NA:Keyword | mobileOG_000719989 | stability/transfer/defense | ICE | Keyword Search | 94.8 | 3435.2 | 0 | 535668 # 541103 # 1 |
| mobileOG_000719988_contig_1 | NA:Keyword | mobileOG_000719988 | transfer | ICE | Keyword Search | 84.7 | 446.8 | 2.5e-124 | 541130 # 541954 # -1 |
| mobileOG_000719987_contig_1 | NA:Keyword | mobileOG_000719987 | transfer | ICE | Keyword Search | 96.8 | 1300.4 | 0 | 558681 # 560681 # -1 |
| mobileOG_000268336_contig_1 | mobB | mobileOG_000268336 | transfer | ICE | Homology | 92.8 | 792.7 | 2.9e-228 | 560784 # 562076 # -1 |
| mobileOG_000341701_contig_1 | traA | mobileOG_000341701 | transfer | Plasmid RefSeq | Homology | 62.7 | 317 | 2.8e-85 | 563252 # 564019 # 1 |
| mobileOG_000719984_contig_1 | NA:Keyword | mobileOG_000719984 | transfer | ICE | Keyword Search | 88.6 | 340.9 | 1.49e-92 | 564775 # 565407 # 1 |
| mobileOG_000341152_contig_1 | traE_1 | mobileOG_000341152 | transfer | Multiple | Homology | 97 | 190.7 | 1.2e-47 | 568954 # 569259 # 1 |
| mobileOG_000268333_contig_1 | traG | mobileOG_000268333 | transfer | ICE | Homology | 99.4 | 1662.9 | 0 | 569601 # 572102 # 1 |
| mobileOG_000268332_contig_1 | traI | mobileOG_000268332 | transfer | ICE | Homology | 100 | 327.8 | 1.3e-88 | 572130 # 572762 # 1 |
| mobileOG_000268331_contig_1 | traJ | mobileOG_000268331 | transfer | ICE | Homology | 99.7 | 634.8 | 7.79e-181 | 572765 # 573760 # 1 |
| mobileOG_000022713_contig_1 | traK | mobileOG_000022713 | transfer | ICE | Manual | 100 | 411.4 | 8.8e-114 | 573785 # 574408 # 1 |
| mobileOG_000022712_contig_1 | traM | mobileOG_000022712 | transfer | ICE | Manual | 99.8 | 868.6 | 4.29e-251 | 575146 # 576486 # 1 |
| mobileOG_000268330_contig_1 | traN | mobileOG_000268330 | transfer | ICE | Homology | 98 | 586.3 | 2.9e-166 | 576512 # 577414 # 1 |
| mobileOG_000719983_contig_1 | NA:Keyword | mobileOG_000719983 | transfer | ICE | Keyword Search | 95.2 | 367.5 | 1.3e-100 | 577436 # 577996 # 1 |
| mobileOG_000268329_contig_1 | traQ | mobileOG_000268329 | transfer | ICE | Homology | 96 | 289.7 | 2.8e-77 | 578008 # 578457 # 1 |
| mobileOG_000022711_contig_1 | gp56 | mobileOG_000022711 | phage | ICE | Manual | 98.3 | 706.8 | 1.7e-202 | 585427 # 586500 # -1 |
| mobileOG_000719981_contig_1 | NA:Keyword | mobileOG_000719981 | integration/excision | ICE | Keyword Search | 96.4 | 804.3 | 9.3e-232 | 587591 # 588841 # -1 |
| mobileOG_000203221_contig_2 | ung | mobileOG_000203221 | replication/recombination/repair | GPD | Homology | 64.1 | 303.5 | 2.8e-81 | 131713 # 132381 # 1 |
| mobileOG_000286654_contig_2 | soj_2 | mobileOG_000286654 | replication/recombination/repair | Multiple | Homology | 63.4 | 325.9 | 6.0e-88 | 211750 # 212523 # -1 |
| mobileOG_000295194_contig_2 | dinG | mobileOG_000295194 | replication/recombination/repair | Plasmid RefSeq | Homology | 64.3 | 336.7 | 3.3e-91 | 297095 # 297853 # -1 |
| mobileOG_000745651_contig_2 | NA:Keyword | mobileOG_000745651 | phage | Multiple | Keyword Search | 60 | 276.6 | 3.8e-73 | 404738 # 405436 # 1 |
| mobileOG_000174406_contig_2 | clpP | mobileOG_000174406 | phage | GPD | Homology | 62.7 | 263.1 | 4.1e-69 | 411571 # 412236 # 1 |
| mobileOG_000251415_contig_2 | cas2 | mobileOG_000251415 | stability/transfer/defense | GPD | Homology | 69.4 | 162.9 | 2.99e-39 | 537275 # 537613 # -1 |
| mobileOG_000346932_contig_2 | cas1 | mobileOG_000346932 | stability/transfer/defense | Plasmid RefSeq | Homology | 62.3 | 387.1 | 2.5e-106 | 537610 # 538500 # -1 |
| mobileOG_000301007_contig_2 | pilT | mobileOG_000301007 | transfer | Plasmid RefSeq | Homology | 63.1 | 77.4 | 9.0e-14 | 545519 # 545704 # 1 |
| mobileOG_000346895_contig_3 | recQ | mobileOG_000346895 | replication/recombination/repair | Plasmid RefSeq | Homology | 65.7 | 986.5 | 2.29e-286 | 68132 # 70333 # -1 |
| mobileOG_000289508_contig_3 | thyA | mobileOG_000289508 | phage | Plasmid RefSeq | Homology | 75.8 | 429.5 | 4.0e-119 | 82681 # 83475 # -1 |
| mobileOG_000008762_contig_3 | gyrB | mobileOG_000008762 | replication/recombination/repair | GPD | Manual | 70.2 | 898.7 | 5.70e-260 | 88184 # 90127 # 1 |
| mobileOG_000346885_contig_3 | parD | mobileOG_000346885 | stability/transfer/defense | Plasmid RefSeq | Homology | 72.5 | 113.6 | 1.49e-24 | 90513 # 90764 # -1 |
| mobileOG_000006286_contig_3 | dnaX | mobileOG_000006286 | replication/recombination/repair | GPD | Manual | 62.7 | 457.6 | 1.9e-127 | 121427 # 122515 # 1 |
| mobileOG_000089584_contig_3 | 35 | mobileOG_000089584 | replication/recombination/repair | GPD | Homology | 60.4 | 182.2 | 6.2e-45 | 132951 # 133394 # 1 |
| mobileOG_000346898_contig_3 | exoA_2 | mobileOG_000346898 | replication/recombination/repair | Plasmid RefSeq | Homology | 63 | 363.6 | 2.6e-99 | 138770 # 139534 # -1 |
| mobileOG_000299805_contig_3 | rarA | mobileOG_000299805 | replication/recombination/repair | Plasmid RefSeq | Homology | 65.1 | 539.7 | 4.4e-152 | 150790 # 152070 # 1 |
| mobileOG_000355646_contig_3 | groL | mobileOG_000355646 | phage | Plasmid RefSeq | Homology | 83.9 | 864 | 1.30e-249 | 179936 # 181561 # -1 |
| mobileOG_000304916_contig_3 | groS | mobileOG_000304916 | phage | Plasmid RefSeq | Homology | 83.1 | 147.9 | 8.09e-35 | 181651 # 181929 # -1 |
| mobileOG_000353838_contig_3 | gyrA | mobileOG_000353838 | replication/recombination/repair | Plasmid RefSeq | Homology | 75.9 | 1241.5 | 0 | 227220 # 229793 # -1 |
| mobileOG_000008241_contig_3 | clpC | mobileOG_000008241 | phage | GPD | Manual | 60.6 | 972.6 | 4.0e-282 | 247492 # 250017 # 1 |
| mobileOG_000208375_contig_3 | rnhB | mobileOG_000208375 | replication/recombination/repair | GPD | Homology | 66.1 | 265.8 | 5.6e-70 | 306299 # 306883 # -1 |
| mobileOG_000055286_contig_5 | tag | mobileOG_000055286 | replication/recombination/repair | COMPASS | Homology | 62.6 | 247.7 | 1.5e-64 | 5028 # 5582 # -1 |
| mobileOG_000013742_contig_5 | clpX | mobileOG_000013742 | phage | GPD | Manual | 64.2 | 482.3 | 7.5e-135 | 31315 # 32463 # -1 |
| mobileOG_000102696_contig_5 | clpP | mobileOG_000102696 | phage | GPD | Homology | 72 | 302 | 8.0e-81 | 32692 # 33357 # -1 |
| mobileOG_000354071_contig_5 | ruvB | mobileOG_000354071 | replication/recombination/repair | Plasmid RefSeq | Homology | 72.1 | 483.4 | 3.0e-135 | 72367 # 73404 # 1 |
| mobileOG_000281926_contig_6 | dnaK | mobileOG_000281926 | phage | Plasmid RefSeq | Homology | 77.6 | 920.2 | 1.8e-266 | 26864 # 28765 # 1 |
| mobileOG_000719990_contig_6 | NA:Keyword | mobileOG_000719990 | transfer | ICE | Keyword Search | 77.6 | 452.6 | 4.7e-126 | 128078 # 128923 # 1 |
| mobileOG_000268337_contig_6 | topB_1 | mobileOG_000268337 | integration/excision | ICE | Homology | 86.9 | 1215.7 | 0 | 131394 # 133478 # 1 |
| mobileOG_000719989_contig_6 | NA:Keyword | mobileOG_000719989 | stability/transfer/defense | ICE | Keyword Search | 90.2 | 3248.8 | 0 | 135158 # 140587 # 1 |
| mobileOG_000719987_contig_6 | NA:Keyword | mobileOG_000719987 | transfer | ICE | Keyword Search | 94 | 1262.3 | 0 | 162197 # 164200 # -1 |
| mobileOG_000268336_contig_6 | mobB | mobileOG_000268336 | transfer | ICE | Homology | 87.2 | 742.3 | 4.5e-213 | 164303 # 165589 # -1 |
| mobileOG_000341701_contig_6 | traA | mobileOG_000341701 | transfer | Plasmid RefSeq | Homology | 62.7 | 322 | 8.6e-87 | 166857 # 167624 # 1 |
| mobileOG_000719984_contig_6 | NA:Keyword | mobileOG_000719984 | transfer | ICE | Keyword Search | 75.6 | 257.7 | 1.7e-67 | 168662 # 169303 # 1 |
| mobileOG_000341152_contig_6 | traE_1 | mobileOG_000341152 | transfer | Multiple | Homology | 96 | 188 | 7.8e-47 | 173144 # 173449 # 1 |
| mobileOG_000294029_contig_6 | traG | mobileOG_000294029 | transfer | Plasmid RefSeq | Homology | 97.1 | 1632.8 | 0 | 173790 # 176294 # 1 |
| mobileOG_000268332_contig_6 | traI | mobileOG_000268332 | transfer | ICE | Homology | 90.5 | 301.6 | 1.0e-80 | 176322 # 176954 # 1 |
| mobileOG_000294028_contig_6 | traJ | mobileOG_000294028 | transfer | Plasmid RefSeq | Homology | 96.8 | 595.9 | 4.0e-169 | 176957 # 177952 # 1 |
| mobileOG_000294027_contig_6 | traK | mobileOG_000294027 | transfer | Plasmid RefSeq | Homology | 97.1 | 403.3 | 2.4e-111 | 177974 # 178597 # 1 |
| mobileOG_000022712_contig_6 | traM | mobileOG_000022712 | transfer | ICE | Manual | 85.5 | 726.5 | 2.69e-208 | 179333 # 180673 # 1 |
| mobileOG_000268330_contig_6 | traN | mobileOG_000268330 | transfer | ICE | Homology | 91 | 550.4 | 1.8e-155 | 180760 # 181659 # 1 |
| mobileOG_000719983_contig_6 | NA:Keyword | mobileOG_000719983 | transfer | ICE | Keyword Search | 92.5 | 362.5 | 4.2e-99 | 181680 # 182240 # 1 |
| mobileOG_000294024_contig_6 | traQ | mobileOG_000294024 | transfer | Plasmid RefSeq | Homology | 94.2 | 265.8 | 4.0e-70 | 182252 # 182668 # 1 |
| mobileOG_000294023_contig_6 | gp56 | mobileOG_000294023 | phage | Plasmid RefSeq | Homology | 96.1 | 689.1 | 3.8e-197 | 197175 # 198248 # -1 |
| mobileOG_000719981_contig_6 | NA:Keyword | mobileOG_000719981 | integration/excision | ICE | Keyword Search | 89.1 | 743.4 | 2.0e-213 | 199334 # 200587 # -1 |
| mobileOG_000019450_contig_7 | hupB | mobileOG_000019450 | phage | GPD | Manual | 71.1 | 115.5 | 4.6e-25 | 86584 # 86871 # 1 |
| mobileOG_000352734_contig_7 | uvrA | mobileOG_000352734 | replication/recombination/repair | Plasmid RefSeq | Homology | 66 | 1248.4 | 0 | 130663 # 133467 # -1 |
| mobileOG_000022711_contig_7 | gp56 | mobileOG_000022711 | phage | ICE | Manual | 86.3 | 627.1 | 1.8e-178 | 179001 # 180074 # 1 |
| mobileOG_000294024_contig_7 | traQ | mobileOG_000294024 | transfer | Plasmid RefSeq | Homology | 67.2 | 176.4 | 3.29e-43 | 180864 # 181298 # -1 |
| mobileOG_000719983_contig_7 | NA:Keyword | mobileOG_000719983 | transfer | ICE | Keyword Search | 60.2 | 244.6 | 1.3e-63 | 181309 # 181872 # -1 |
| mobileOG_000294025_contig_7 | traN | mobileOG_000294025 | transfer | Plasmid RefSeq | Homology | 64 | 382.9 | 4.79e-105 | 181883 # 182776 # -1 |
| mobileOG_000294027_contig_7 | traK | mobileOG_000294027 | transfer | Plasmid RefSeq | Homology | 79.7 | 346.7 | 2.7e-94 | 184426 # 185049 # -1 |
| mobileOG_000294028_contig_7 | traJ | mobileOG_000294028 | transfer | Plasmid RefSeq | Homology | 81.8 | 538.5 | 7.6e-152 | 185071 # 186063 # -1 |
| mobileOG_000268332_contig_7 | traI | mobileOG_000268332 | transfer | ICE | Homology | 83.3 | 265.4 | 8.5e-70 | 186066 # 186743 # -1 |
| mobileOG_000294029_contig_7 | traG | mobileOG_000294029 | transfer | Plasmid RefSeq | Homology | 90.1 | 1532.7 | 0 | 186776 # 189271 # -1 |
| mobileOG_000294030_contig_7 | traF | mobileOG_000294030 | transfer | Plasmid RefSeq | Homology | 76 | 158.7 | 5.5e-38 | 189268 # 189600 # -1 |
| mobileOG_000341152_contig_7 | traE_1 | mobileOG_000341152 | transfer | Multiple | Homology | 88.1 | 176.4 | 2.3e-43 | 189611 # 189916 # -1 |
| mobileOG_000268335_contig_7 | traA | mobileOG_000268335 | transfer | ICE | Homology | 76.1 | 389.8 | 3.4e-107 | 193943 # 194710 # -1 |
| mobileOG_000268336_contig_7 | mobB | mobileOG_000268336 | transfer | ICE | Homology | 61.8 | 518.1 | 1.4e-145 | 195802 # 197088 # 1 |
| mobileOG_000719987_contig_7 | NA:Keyword | mobileOG_000719987 | transfer | ICE | Keyword Search | 84.4 | 1120.9 | 0 | 197190 # 199157 # 1 |
| mobileOG_000208799_contig_8 | clpB | mobileOG_000208799 | phage | GPD | Homology | 62.6 | 911.8 | 8.69e-264 | 29012 # 31624 # 1 |
| mobileOG_000345422_contig_8 | ruvC | mobileOG_000345422 | replication/recombination/repair | Plasmid RefSeq | Homology | 68.1 | 254.6 | 1.2e-66 | 117301 # 117849 # 1 |
| mobileOG_000282641_contig_9 | recA | mobileOG_000282641 | replication/recombination/repair | Plasmid RefSeq | Homology | 82.9 | 550.4 | 2.1e-155 | 10819 # 11880 # -1 |
| mobileOG_000299804_contig_9 | hup | mobileOG_000299804 | phage | Plasmid RefSeq | Homology | 62.6 | 119 | 4.3e-26 | 57321 # 57617 # -1 |
| mobileOG_000173697_contig_9 | dut | mobileOG_000173697 | transfer | GPD | Homology | 71.1 | 206.1 | 3.9e-52 | 111722 # 112156 # -1 |
| mobileOG_000112466_contig_9 | rnhA | mobileOG_000112466 | replication/recombination/repair | GPD | Homology | 62.9 | 179.1 | 5.6e-44 | 130696 # 131169 # 1 |
| mobileOG_000719990_contig_11 | NA:Keyword | mobileOG_000719990 | transfer | ICE | Keyword Search | 64.7 | 377.1 | 2.5e-103 | 50145 # 50996 # 1 |
| mobileOG_000013745_contig_12 | uvrB | mobileOG_000013745 | replication/recombination/repair | GPD | Manual | 65.7 | 864 | 1.6e-249 | 8281 # 10272 # -1 |
| mobileOG_000355105_contig_15 | uvrA | mobileOG_000355105 | replication/recombination/repair | Plasmid RefSeq | Homology | 65.5 | 1262.3 | 0 | 19891 # 22758 # -1 |
| mobileOG_000299815_contig_17 | topB | mobileOG_000299815 | replication/recombination/repair | Plasmid RefSeq | Homology | 66.2 | 908.7 | 6.0e-263 | 18297 # 20438 # -1 |
| mobileOG_000327613_contig_18 | radA | mobileOG_000327613 | replication/recombination/repair | Plasmid RefSeq | Homology | 65.4 | 592.8 | 4.7e-168 | 24060 # 25424 # 1 |
| mobileOG_000725006_contig_19 | NA:Keyword | mobileOG_000725006 | integration/excision | ISFinder | Keyword Search | 63.3 | 122.1 | 5.6e-27 | 37739 # 38065 # -1 |
| mobileOG_000310209_contig_20 | gin | mobileOG_000310209 | integration/excision | Multiple | Homology | 71 | 293.1 | 3.5e-78 | 24842 # 25459 # -1 |
| mobileOG_000734222_contig_20 | NA:Keyword | mobileOG_000734222 | replication/recombination/repair | Plasmid RefSeq | Keyword Search | 64.8 | 434.9 | 1.2e-120 | 30597 # 31610 # 1 |
| mobileOG_000026128_contig_21 | dinB | mobileOG_000026128 | replication/recombination/repair | Multiple | Manual | 62.8 | 440.7 | 2.3e-122 | 16386 # 17465 # -1 |
| mobileOG_000320449_contig_30 | binR | mobileOG_000320449 | integration/excision | Plasmid RefSeq | Homology | 63.6 | 59.3 | 1.8e-08 | 569 # 703 # 1 |
| mobileOG_000271717_contig_35 | ORFC | mobileOG_000271717 | integration/excision | immedb | Homology | 66.5 | 282 | 7.79e-75 | 3 # 605 # -1 |

Supplemental Table 3. Predicted virulence factors

|  |  |  |  |  |  |  |
| --- | --- | --- | --- | --- | --- | --- |
| **VF class** | **Virulence factors** | **Related genes** | **E. brevis GBW-1** | **GCF_008705155.1 (E. brevis SE1-3)** | **GCF_009834475.1 (E. brevis BCLYD2)** |  |
|  |  |  | **draft (Dec_14-7080580523;draft)** | **NZ_CP043634.1 (Dec_25-1145736748;NZ_CP043634.1)** | **NZ_CP013210.1 (Dec_25-4202234959;NZ_CP013210.1)** |  |
| Adherence | Flp type IV pili | *flp1* | - | - | - |  |
|  |  | *flpA* | - | - | - |  |
|  |  | *flpB* | - | - | - |  |
|  |  | *flpC* | - | - | - |  |
|  |  | *flpD* | - | - | - |  |
|  |  | *flpE* | - | - | - |  |
|  |  | *flpF* | - | - | - |  |
|  |  | *flpG* | - | - | - |  |
|  |  | *flpH* | - | - | - |  |
|  |  | *flpI* | - | - | - |  |
|  |  | *flpJ* | - | - | - |  |
|  |  | *flpK* | - | - | - |  |
|  |  | *flpL* | - | - | - |  |
|  | Lateral flagella | *flgC* | - | - | - |  |
|  |  | *flgE* | - | - | - |  |
|  |  | *flgI* | - | - | - |  |
|  |  | *flgJ* | - | - | - |  |
|  |  | *fliF* | - | - | - |  |
|  |  | *fliG* | - | - | - |  |
|  |  | *fliP* | - | - | - |  |
|  |  | *lafB* | - | - | - |  |
|  |  | *lafC* | - | - | - |  |
|  |  | *lafE* | - | - | - |  |
|  |  | *lafF* | - | - | - |  |
|  |  | *lafK* | - | - | - |  |
|  |  | *lafS* | - | - | - |  |
|  |  | *lafT* | - | - | - |  |
|  |  | *lafU* | - | - | - |  |
|  |  | *lafX* | - | - | - |  |
|  |  | *lfgA* | - | - | - |  |
|  |  | *lfgB* | - | - | - |  |
|  |  | *lfgF* | - | - | - |  |
|  |  | *lfgG* | - | - | - |  |
|  |  | *lfgH* | - | - | - |  |
|  |  | *lfgK* | - | - | - |  |
|  |  | *lfgL* | - | - | - |  |
|  |  | *lfgM* | - | - | - |  |
|  |  | *lfgN* | - | - | - |  |
|  |  | *lfhA* | - | - | - |  |
|  |  | *lfhB* | - | - | - |  |
|  |  | *lfiE* | - | - | - |  |
|  |  | *lfiH* | - | - | - |  |
|  |  | *lfiI* | - | - | - |  |
|  |  | *lfiJ* | - | - | - |  |
|  |  | *lfiM* | - | - | - |  |
|  |  | *lfiN* | - | - | - |  |
|  |  | *lfiQ* | - | - | - |  |
|  |  | *lfiR* | - | - | - |  |
|  |  | *maf-5* | - | - | - |  |
|  | Mannose-sensitive hemagglutinin (Msh) pilus | *mshA* | - | - | - |  |
|  |  | *mshB* | - | - | - |  |
|  |  | *mshC* | - | - | - |  |
|  |  | *mshD* | - | - | - |  |
|  |  | *mshE* | - | - | - |  |
|  |  | *mshF* | - | - | - |  |
|  |  | *mshG* | - | - | - |  |
|  |  | *mshI1* | - | - | - |  |
|  |  | *mshI* | - | - | - |  |
|  |  | *mshJ* | - | - | - |  |
|  |  | *mshK* | - | - | - |  |
|  |  | *mshL* | - | - | - |  |
|  |  | *mshM* | - | - | - |  |
|  |  | *mshN* | - | - | - |  |
|  |  | *mshO* | - | - | - |  |
|  |  | *mshP* | - | - | - |  |
|  |  | *mshQ* | - | - | - |  |
|  | Polar flagella | *Undetermined* | - | - | - |  |
|  |  | *Undetermined* | - | - | - |  |
|  |  | *cheA-2* | - | - | - |  |
|  |  | *cheB-2* | - | - | - |  |
|  |  | *cheR-3* | - | - | - |  |
|  |  | *cheV* | - | - | - |  |
|  |  | *cheW* | - | - | - |  |
|  |  | *cheY* | - | - | - |  |
|  |  | *cheZ* | - | - | - |  |
|  |  | *flaA* | - | - | - |  |
|  |  | *flaB* | - | - | - |  |
|  |  | *flaG* | - | - | - |  |
|  |  | *flaH* | - | - | - |  |
|  |  | *flaJ* | - | - | - |  |
|  |  | *flgA* | - | - | - |  |
|  |  | *flgB* | - | - | - |  |
|  |  | *flgC* | - | - | - |  |
|  |  | *flgD* | - | - | - |  |
|  |  | *flgE* | - | - | - |  |
|  |  | *flgF* | - | - | - |  |
|  |  | *flgG* | - | - | - |  |
|  |  | *flgH* | - | - | - |  |
|  |  | *flgI* | - | - | - |  |
|  |  | *flgJ* | - | - | - |  |
|  |  | *flgK* | - | - | - |  |
|  |  | *flgL* | - | - | - |  |
|  |  | *flgM* | - | - | - |  |
|  |  | *flgN* | - | - | - |  |
|  |  | *flhA* | - | - | - |  |
|  |  | *flhB* | - | - | - |  |
|  |  | *flhF* | - | - | - |  |
|  |  | *flhG* | - | - | - |  |
|  |  | *fliA* | - | - | - |  |
|  |  | *fliE* | - | - | - |  |
|  |  | *fliF* | - | - | - |  |
|  |  | *fliG* | - | - | - |  |
|  |  | *fliH* | - | - | - |  |
|  |  | *fliI* | - | - | - |  |
|  |  | *fliJ* | - | - | - |  |
|  |  | *fliK* | - | - | - |  |
|  |  | *fliL* | - | - | - |  |
|  |  | *fliM* | - | - | - |  |
|  |  | *fliN* | - | - | - |  |
|  |  | *fliO* | - | - | - |  |
|  |  | *fliP* | - | - | - |  |
|  |  | *fliQ* | - | - | - |  |
|  |  | *fliR* | - | - | - |  |
|  |  | *flmD* | - | - | - |  |
|  |  | *flmH* | - | - | - |  |
|  |  | *flrA* | - | - | - |  |
|  |  | *flrB* | - | - | - |  |
|  |  | *flrC* | - | - | - |  |
|  |  | *maf-1* | - | - | - |  |
|  |  | *maf-2* | - | - | - |  |
|  |  | *motX* | - | - | - |  |
|  |  | *motY* | - | - | - |  |
|  |  | *nueA* | - | - | - |  |
|  |  | *nueB* | - | - | - |  |
|  |  | *pomA2* | - | - | - |  |
|  |  | *pomA* | - | - | - |  |
|  |  | *pomB2* | - | - | - |  |
|  |  | *pomB* | - | - | - |  |
|  | Tap type IV pili | *tapA* | - | - | - |  |
|  |  | *tapB* | - | - | - |  |
|  |  | *tapC* | - | - | - |  |
|  |  | *tapD* | - | - | - |  |
|  |  | *tapF* | - | - | - |  |
|  |  | *tapM* | - | - | - |  |
|  |  | *tapN* | - | - | - |  |
|  |  | *tapO* | - | - | - |  |
|  |  | *tapP* | - | - | - |  |
|  |  | *tapQ* | - | - | - |  |
|  |  | *tapT* | - | - | - |  |
|  |  | *tapU* | - | - | - |  |
|  |  | *tapV* | - | - | - |  |
|  |  | *tapW* | - | - | - |  |
|  |  | *tapY1* | - | - | - |  |
|  |  | *tapY2* | - | - | - |  |
|  |  | *tppA* | - | - | - |  |
|  |  | *tppB* | - | - | - |  |
|  |  | *tppC* | - | - | - |  |
|  |  | *tppD* | - | - | - |  |
|  |  | *tppE* | - | - | - |  |
|  |  | *tppF* | - | - | - |  |
|  | Type I fimbriae | *fimA* | - | - | - |  |
|  |  | *fimC* | - | - | - |  |
|  |  | *fimD* | - | - | - |  |
|  |  | *fimE* | - | - | - |  |
|  |  | *fimF* | - | - | - |  |
|  | GroEL(Clostridium) | *groEL* | orf01346 | orf01492 | orf01153 |  |
|  | LPS O-antigen (P. aeruginosa) |  | orf03304 | orf00827 | - |  |
| Secretion system | T2SS | *exeA* | - | - | - |  |
|  |  | *exeB* | - | - | - |  |
|  |  | *exeC* | - | - | - |  |
|  |  | *exeD* | - | - | - |  |
|  |  | *exeE* | - | - | - |  |
|  |  | *exeF* | - | - | - |  |
|  |  | *exeG* | - | - | - |  |
|  |  | *exeH* | - | - | - |  |
|  |  | *exeI* | - | - | - |  |
|  |  | *exeJ* | - | - | - |  |
|  |  | *exeK* | - | - | - |  |
|  |  | *exeL* | - | - | - |  |
|  |  | *exeM* | - | - | - |  |
|  |  | *exeN* | - | - | - |  |
|  |  | *tapD* | - | - | - |  |
|  | T3SS | *acr1* | - | - | - |  |
|  |  | *acr2* | - | - | - |  |
|  |  | *acrG* | - | - | - |  |
|  |  | *acrH* | - | - | - |  |
|  |  | *acrR* | - | - | - |  |
|  |  | *acrV* | - | - | - |  |
|  |  | *aexT* | - | - | - |  |
|  |  | *aopB* | - | - | - |  |
|  |  | *aopD* | - | - | - |  |
|  |  | *aopH* | - | - | - |  |
|  |  | *aopN* | - | - | - |  |
|  |  | *aopO* | - | - | - |  |
|  |  | *aopX* | - | - | - |  |
|  |  | *ascB* | - | - | - |  |
|  |  | *ascC* | - | - | - |  |
|  |  | *ascD* | - | - | - |  |
|  |  | *ascE* | - | - | - |  |
|  |  | *ascF* | - | - | - |  |
|  |  | *ascG* | - | - | - |  |
|  |  | *ascH* | - | - | - |  |
|  |  | *ascI* | - | - | - |  |
|  |  | *ascJ* | - | - | - |  |
|  |  | *ascK* | - | - | - |  |
|  |  | *ascL* | - | - | - |  |
|  |  | *ascN* | - | - | - |  |
|  |  | *ascO* | - | - | - |  |
|  |  | *ascP* | - | - | - |  |
|  |  | *ascQ* | - | - | - |  |
|  |  | *ascR* | - | - | - |  |
|  |  | *ascS* | - | - | - |  |
|  |  | *ascT* | - | - | - |  |
|  |  | *ascU* | - | - | - |  |
|  |  | *ascV* | - | - | - |  |
|  |  | *ascX* | - | - | - |  |
|  |  | *ascY* | - | - | - |  |
|  |  | *ati1* | - | - | - |  |
|  |  | *ati2* | - | - | - |  |
|  |  | *exsA* | - | - | - |  |
|  |  | *exsB* | - | - | - |  |
|  |  | *exsC* | - | - | - |  |
|  |  | *exsD* | - | - | - |  |
|  |  | *exsE* | - | - | - |  |
|  |  | *sycH* | - | - | - |  |
|  |  | *sycO* | - | - | - |  |
|  |  | *sycX* | - | - | - |  |
|  | T6SS | *Undetermined* | - | - | - |  |
|  |  | *atsA* | - | - | - |  |
|  |  | *atsB* | - | - | - |  |
|  |  | *atsC* | - | - | - |  |
|  |  | *atsD* | - | - | - |  |
|  |  | *atsG* | - | - | - |  |
|  |  | *atsH* | - | - | - |  |
|  |  | *atsI* | - | - | - |  |
|  |  | *atsJ* | - | - | - |  |
|  |  | *atsK* | - | - | - |  |
|  |  | *atsL* | - | - | - |  |
|  |  | *atsP* | - | - | - |  |
|  |  | *atsQ* | - | - | - |  |
|  |  | *atsS* | - | - | - |  |
|  |  | *clpV1* | orf01407 | orf01553 | orf01216 |  |
|  |  | *dotU* | - | - | - |  |
|  |  | *hcp1* | - | - | - |  |
|  |  | *hcp* | - | - | - |  |
|  |  | *vasH* | - | - | - |  |
|  |  | *vasK/atsR* | - | - | - |  |
|  |  | *vgrG1* | - | - | - |  |
|  |  | *vgrG2* | - | - | - |  |
|  |  | *vgrG3* | - | - | - |  |
|  |  | *vipA* | - | - | - |  |
|  |  | *vipB* | - | - | - |  |
|  | T4SS effectors(Coxiella) |  | orf00046 | orf00173 | orf03476 |  |
|  | T6SS-II (Klebsiella) |  | orf02426 | orf01061 | orf00721 |  |
| Toxin | Aerolysin AerA/Cytotoxic enterotoxin Act | *aerA/act* | - | - | - |  |
|  | Extracellular hemolysin AHH1 | *ahh1* | - | - | - |  |
|  | Heat-stable cytotonic enterotoxin | *ast* | - | - | - |  |
|  | Hemolysin HlyA | *hlyA* | - | - | - |  |
|  | Hemolysin III | *Undetermined* | - | - | - |  |
|  | The repeat in toxin (RTX) | *rtxA* | - | - | - |  |
|  |  | *rtxB* | - | - | - |  |
|  |  | *rtxC* | - | - | - |  |
|  |  | *rtxD* | - | - | - |  |
|  |  | *rtxE* | - | - | - |  |
|  |  | *rtxH* | - | - | - |  |
|  | Thermostable hemolysin (TH) | *Undetermined* | - | - | - |  |
| Adherence and invasion | EF-Tu(Francisella) |  | orf02733 | orf00505 | orf00178 |  |
| Antiphagocytosis | Capsule I(Burkholderia) |  | - | - | orf00529 |  |
|  | Capsule(Klebsiella) | *uge* | orf03308 | orf00831 | orf00498 |  |
| Enzyme | Streptococcal enolase(Streptococcus) | *eno* | orf00007 | orf00211 | orf03515 |  |
| Glycosylation system | N-linked protein glycosylation(Campylobacter) | *pglC* | orf03473 | orf00816 | - |  |
| Immune evasion | Capsule(Acinetobacter) |  | orf03305 | orf00474; orf00826; orf00828 | orf00145; orf00494; orf00495 |  |
|  | Capsule(Staphylococcus) | *capL* | orf03477 | orf00812 | orf00456 |  |
|  | Capsule(Streptococcus) | *rfbA-1* | - | - | orf00496 |  |
|  |  | *rmlA* | orf03306 | orf00829 | - |  |
|  | Exopolysaccharide (Haemophilus) | *pgi* | orf02655 | orf00789 | orf00433 |  |
|  | LPS(Brucella) | *acpXL* | orf03315 | orf00838 | orf00505 |  |
| Iron uptake | Acinetobactin (Acinetobacter) | *bauE* | orf00565 | orf03156 | orf02901 |  |
|  | Heme biosynthesis(Haemophilus) | *hemL* | orf01117 | orf02701 | orf02484 |  |
| Lipid and fatty acid metabolism | Isocitrate lyase(Mycobacterium) | *icl* | orf02997 | orf02124 | orf01882 |  |
| Stress adaptation | Catalase-peroxidase(Mycobacterium) | *katG* | orf00189 | orf00049 | orf03333 |  |

Supplemental Table 4. Characteristics of EBR variants

| **Variant** | **Host species** | **Source** | **Location** | **Reference** |
| --- | --- | --- | --- | --- |
| **EBR-1** | *E. brevis* | Clinical-rectal swab | Le-Kremlin-Bicetre, France | [1] |
| **EBR-2** | *E. falsenii* | Clinical-ear swab | Buenos, Aires, Argentina | [2] |
| **EBR-3** | *E. falsenii* | Clinical-rectal swab | France | NG_066507 |
| **EBR-4** | *E. falsenii* | Clinical-Stool | Nigeria | [3] |
| **EBR-5** | *E. stercoris* | Chicken-rectal swab | Chongqing, China | [4] |
| **EBR-6** | *E. brevis* | Ground beef | Wisconsin, USA | This study |

Supplemental Table 5. Identification and classification of carbapenemase from *E. brevis* GBW-1

| Strain | mCIM (mm) | eCIM (mm) |
| --- | --- | --- |
| MBL Positive control *Pseudomonas aeruginosa* ARB0103 (*bla*_IMP1+_) | 6 | 21 |
| MBL Negative control *Enterobacter cloacae* ARB0053 (*bla*_KPC3+_ ) | 6 | 6 |
| *Empedobacter brevis* GBW-1 | 20 | 25 |

MBL: metallo-beta-lactamase

Supplemental Figure

Supplemental Table 6. Predicted docking binding energies of EBR to meropenem

| **Protein** | **Mode** | **Affinity (Kcal/mol)** | **Distance from best mode** | |
| --- | --- | --- | --- | --- |
|  |  |  | **RMSD l.b.** | **RMSD u.b.** |
| **EBR-1** | 1 | -6.6 | 0.000 | 0.000 |
|  | 2 | -6.5 | 11.331 | 14.489 |
|  | 3 | -6.5 | 1.835 | 2.355 |
|  | 4 | -6.4 | 2.399 | 8.360 |
|  | 5 | -6.3 | 3.242 | 8.194 |
|  | 6 | -6.1 | 3.712 | 5.135 |
|  | 7 | -5.7 | 10.503 | 14.371 |
|  | 8 | -5.5 | 2.601 | 7.772 |
|  | 9 | -5.4 | 10.318 | 13.965 |
|  | | | | |
| **EBR-5** | 1 | -7.5 | 0.000 | 0.000 |
|  | 2 | -7.2 | 3.473 | 4.097 |
|  | 3 | -7.0 | 3.825 | 5.466 |
|  | 4 | -6.2 | 21.442 | 24.821 |
|  | 5 | -6.2 | 4.394 | 5.304 |
|  | 6 | -5.8 | 5.120 | 7.680 |
|  | 7 | -5.6 | 4.688 | 7.137 |
|  | 8 | -5.4 | 25.701 | 28.124 |
|  | 9 | -5.4 | 27.990 | 30.421 |
|  | | | | |
| **EBR-6** | 1 | -7.1 | 0.000 | 0.000 |
|  | 2 | -5.7 | 16.291 | 19.725 |
|  | 3 | -5.4 | 29.297 | 30.155 |
|  | 4 | -5.3 | 27.656 | 28.959 |
|  | 5 | -5.3 | 17.257 | 19.402 |
|  | 6 | -5.2 | 10.912 | 13.742 |
|  | 7 | -5.2 | 22.196 | 24.149 |
|  | 8 | -5.2 | 17.334 | 18.828 |
|  | 9 | -5.1 | 27.701 | 29.650 |

1. Bellais S, Girlich D, Karim A, Nordmann P: **EBR-1, a novel Ambler subclass B1 beta-lactamase from Empedobacter brevis**. *Antimicrob Agents Chemother* 2002, **46**(10):3223-3227.

2. Collins C, Almuzara M, Saigo M, Montaña S, Chiem K, Traglia G, Mussi MA, Tolmasky M, Iriarte A, Vay C *et al*: **Whole-Genome Analysis of an Extensively Drug-Resistance Empedobacter falsenii Strain Reveals Distinct Features and the Presence of a Novel Metallo-ß-Lactamase (EBR-2)**. *Curr Microbiol* 2018, **75**(8):1084-1089.

3. Olowo-Okere A, Ibrahim YKE, Olayinka BO, Mohammed Y, Nabti LZ, Lupande-Mwenebitu D, Rolain JM, Diene SM: **Genomic features of an isolate of Empedobacter falsenii harbouring a novel variant of metallo-beta-lactamase, bla(EBR-4) gene**. *Infect Genet Evol* 2022, **98**:105234.

4. Li P, Lei T, Zhou Y, Dai Y, Yang Z, Luo H: **EBR-5, a Novel Variant of Metallo-beta-Lactamase EBR from Multidrug-Resistant Empedobacter stercoris**. *Microbiol Spectr* 2023, **11**(2):e0003923.
